# Prevalence of asymptomatic bacteriuria in type 2 diabetic subjects with and without microalbuminuria

**DOI:** 10.1186/1756-0500-3-169

**Published:** 2010-06-17

**Authors:** Athanasia Papazafiropoulou, Ioannis Daniil, Alexios Sotiropoulos, Eleni Balampani, Anthi Kokolaki, Stavros Bousboulas, Stavroula Konstantopoulou, Eystathios Skliros, Dimitra Petropoulou, Stavros Pappas

**Affiliations:** 1 3rd Department of Internal Medicine and Center of Diabetes, General Hospital of Nikaia "Ag. Panteleimon" - Piraeus, Greece; 2 Department of Microbiology, General Hospital of Nikaia "Ag. Panteleimon" - Piraeus, Greece

## Abstract

**Background:**

Diabetic subjects, especially women, show high prevalence of asymptomatic bacteriuria (ASB). The aim of the present study was to evaluate the prevalence of ASB in subjects with type 2 diabetes mellitus (T2D) with and without microalbuminuria (MA).

**Findings:**

A hundred diabetic subjects with MA (53 males/47 females, mean age ± standard deviation: 65.5 ± 11.1 years) and 100 diabetic subjects without MA (52 males/48 females, mean age ± standard deviation: 65.4 ± 11.3 years), consecutively attending the outpatient diabetes clinic of our hospital were recruited in the study. Subjects with overt diabetic nephropathy or nephropathy from other causes were excluded. In addition, subjects with symptoms of urinary track infection or use of antimicrobial drugs in the last 14 days were excluded by the study.

Diabetic subjects with MA showed increased prevalence of ASB compared to diabetic subjects without MA (21% versus 8%, P < 0.001, respectively). *Escherichia coli *was the most prevalent pathogen isolated in diabetic subjects with and without MA (12% versus 3.0%, P = 0.01, respectively) followed by *Proteus mirabilis *(6% versus 5%, P = 0.75, respectively) and *Klebsiella *spp (5% versus 1%, P = 0.09, respectively). Univariate logistic analysis showed that ASB was associated with the presence of coronary artery disease [odds ratio (OR): 0.29, 95% Confidence Intervals (95% CI): 0.09-0.95, P = 0.04] and gender (OR: 0.09, 95% CI: 0.02-0.35, P < 0.001) in the diabetic study group with MA.

**Conclusions:**

ASB is more prevalent among T2D subjects with MA. Screening for ASB is warranted in diabetic patients especially if pyuria is detected in urine analysis since ASB has been found to be a risk factor for developing symptomatic urinary tract infection.

## Background

Urinary tract infections (UTIs) as well as their complications, such as emphysematous cystitis, pyelonephritis and renal papillary necrosis occur more commonly in subjects with type 2 diabetes mellitus (T2D) [1-3]. In addition, many studies have showed that diabetic subjects, especially women, show high prevalence of asymptomatic bacteriuria (ASB) [4-7]. In diabetic women, various risk factors for ASB have been suggested including age, presence of macroalbuminuria, low body mass index (BMI) and UTIs during the previous year [7]. In a prior study, ASB was a risk factor for subsequent decline in renal function among women with type 1 diabetes mellitus [8].

Microalbuminuria (MA) is a common complication of T2D affecting almost 30-50% of the patients with T2D [9,10]. Presence of MA has been related with high blood pressure, dyslipidemia, inflammation, endothelial dysfunction, left ventricular hypertrophy and hypercoagulation [11]. However, little evidence exists regarding the relationship between ASB and MA in T2D subjects. Therefore, the aim of the present study was to evaluate the prevalence of ASB in T2D subjects with and without MA and to determine possible risk factors affecting the presence of ASB in diabetic subjects.

## Methods

A hundred diabetic subjects with MA (53 males/47 females, mean age ± standard deviation: 65.5 ± 11.1 years) and 100 diabetic subjects without MA (52 males/48 females, mean age ± standard deviation: 65.4 ± 11.3 years), consecutively attending the outpatient diabetes clinic of our hospital were recruited in the study. The physical examination and the interview of the subjects were carried based on a study protocol by the investigators. Subjects with overt diabetic nephropathy (proteinuria) or nephropathy from other causes were excluded. In addition, subjects with symptoms of UTI (including dysuria, frequency, fever, urgency and abdominal discomfort) or use of antimicrobial drugs in the last 14 days were excluded by the study.

All participants underwent complete physical examination in the morning of the study. They were questioned about previous and current diseases, use of medications and their smoking habits; ex-smokers who had given up smoking for a period of at least three years were considered as non-smokers. Retinopathy was reported from the medical records. Body weight with subjects in light clothing without shoes and height was measured and body mass index (BMI) was calculated. Waist circumference was measured with a soft tape on standing, midway between the lowest rib and the iliac crest.

Blood pressure was measured in the right arm three consecutive times, one minute apart, in the sitting position after 10 min resting period, using an appropriate cuff size. The mean value of the last two measurements was used in the statistical analysis. Arterial hypertension was defined according to the current guidelines if systolic blood pressure was ≥140 mmHg and/or diastolic blood pressure ≥ 90 mmHg or if patients were on antihypertensive treatment [12].

Blood was collected after an overnight fast of at least 12 hours. Serum glucose, lipids [total cholesterol, high density lipoprotein (HDL) cholesterol, triglycerides] and creatinine were measured enzymatically on a Technicon RA-XT analyzer (Technicon Ltd, Dublin, Ireland). Low density lipoprotein (LDL) cholesterol was calculated using the formula of Friedwald et al [13]. Glomerular filtration rate was calculated according to the equation of Cockcroft and Gault [14]. HbA_1c _was measured by HPLC (Roche diagnostics, Mannheim, Germany) with a non-diabetic reference range of 4.1-6.0%. Microalbuminuria was diagnosed when albumin excretion rate (AER), measured by radioimmunoassay (RIA) (Pharmacia, Pharmacia and Upjon Diagnostics AB, Upsala, Sweden), was 30-300 mg/24-hours in at least two out of three 24-hours urine collections over a three month period.

Midstream clean voiding urinary specimens were collected for urinalysis, microscopy, culture and sensitivity. The specimens were refrigerated immediately and cultured within 2 hours. All urine samples were cultured on Blood and MacConkey agar plates. The plates were incubated at 37°C aerobically for 48 hours. Bacteriuria was defined as the presence of at least 10^5 ^colony forming units/ml of 1 or 2 bacterial species in a culture of clean-voided midstream urine confirmed by a second culture. Presence of at least three different microorganisms in a urine specimen was considered as contamination [15].

The study protocol was approved by the Scientific and Ethical Committee of the General Hospital of Nikaia. Full informed written consent was obtained from all patients.

## Statistical analysis

Analyses were performed using the SPSS 15.0 (SPSS, IL, USA) statistical package. All variables were tested for normal distribution of the data. Data are presented as means ± standard deviation or percentages. Differences between the studied groups examined using the student's unpaired *t-*test or the Mann-Whitney *U*-test for parametric and non-parametric data, respectively, while a chi-square test was used for categorical data. Bivariate correlations were performed using the Pearson or the Spearman correlation coefficient, as appropriate. Univariate binary logistic analysis was performed to look for the relationship between ASB and the variables of interest in the sample population. *P *values < 0.05 were considered statistically significant.

## Results

### Subjects characteristics

More diabetic subjects with MA had coronary artery disease (CAD) (39% versus 23%, P < 0.01, respectively) and hypertension (87% versus 72%, P < 0.001, respectively) compared to diabetic subjects without MA. In addition, diabetic subjects with MA had higher levels of plasma urea (P < 0.001) and creatinine (P = 0.03) than diabetic subjects without MA. Patients with and without MA did not differ significantly in terms of age, BMI, duration of diabetes, glycemic control, systolic and diastolic arterial pressure, lipid profile, diabetic complications and treatment for diabetes (Table 1).

**Table 1:** Demographic and clinical characteristics of diabetic subjects with and without microalbuminuria (MA)

	MA (+)	MA (-)	*P*
Males/females	53/47	52/48	0.88
			
Age (years)	65.5 ± 11.1	65.4 ± 11.3	0.94
Waist (cm)	105.5 ± 11.4	104.1 ± 12.5	0.82
Current smokers	45	44	0.88
Duration of diabetes (years)	14.7 ± 7.8	13.6 ± 8.2	0.39
HbA1c (%)	7.6 ± 1.4	8.1 ± 1.4	0.46
Body mass index (Kg/m^2^)	32.7 ± 8.3	31.4 ± 6.2	0.18
Systolic blood pressure (mm Hg)	140.9 ± 18.1	141.2 ± 18.2	0.92
			
Diastolic blood pressure (mm Hg)	75.7 ± 10.2	76.9 ± 12.8	0.47
			
Glucose (mg/dl)	156.8 ± 44.6	155.9 ± 53.8	0.89
Total cholesterol (mg/dl)	178.3 ± 54.9	176.6 ± 38.4	0.81
			
HDL cholesterol (mg/dl)	48.6 ± 14.7	49.9 ± 15.2	0.54
			
LDL cholesterol (mg/dl)	98.8 ± 35.5	101.3 ± 32.7	0.54
			
Triglycerides (mg/dl)	145.4 ± 38.0	133.9 ± 32.9	0.34
			
Urea (mg/dl)	47.2 ± 14.1	37.0 ± 12.7	**< 0.001**
			
Creatinine (mg/dl)	1.1 ± 0.6	0.9 ± 0.3	**0.03**
CAD (yes)	39	23	**0.01**
Retinopathy (yes)	44	39	0.47
Hypertension (yes)	87	72	**< 0.001**
Hyperlipidemia (yes)	74	81	0.24
Treatment for diabetes	-	-	-
Antidiabetic tablets	71	73	0.75
Insulin	63	54	0.75

P values for the comparison between subjects with and without MA by independent samples t-test for continuous variables or by Pearson χ^2 ^for nominal variables.

HDL: high density lipoprotein; LDL: low density lipoprotein; CAD: coronary artery disease; PAD: peripheral artery disease.

### Prevalence of ASB and isolated pathogens

Diabetic subjects with MA showed increased prevalence of ASB compared to diabetic subjects without MA (21% versus 8%, P < 0.001, respectively). *Escherichia coli *was the most prevalent pathogen isolated in diabetic subjects with and without MA (12% versus 3.0%, P = 0.01, respectively) followed by *Proteus mirabilis *(6% versus 5%, P = 0.75, respectively) and *Klebsiella *spp (5% versus 1%, P = 0.09, respectively).

Univariate logistic analysis showed that ASB was associated with the presence of CAD [odds ratio (OR): 0.29, 95% Confidence Intervals (95% CI): 0.09-0.95, P = 0.04] and gender (OR: 0.09, 95% CI: 0.02-0.35, P < 0.001) in the diabetic study group with MA. No any significant relationships were found between ASB and the rest of the variables. The same pattern was observed in diabetic study group without MA (Table 2).

**Table 2:** Univariate logistic analysis: the association between various parameters with ASB in diabetic subjects with and without microalbuminuria (MA)

	MA (+)			MA (-)		
	Odds ratio	95% Confidence Intervals	P-value	Odds ratio	95% Confidence Intervals	P-value
Gender (males vs. females)	0.09	0.02-0.35	**< 0.001**	0.98	0.88-1.17	0.61
						
Age	0.99	0.95-1.04	0.98	1.01	0.94-1.08	0.67
Waist	0.97	0.85-1.02	0.81	1.02	0.98-1.08	0.81
Current smokers	0.89	0.34-2.36	0.82	1.30	0.31-5.51	0.72
Duration of diabetes	0.99	0.95-1.04	0.91	1.05	0.97-1.14	0.19
HbA1c	0.78	0.52-1.16	0.23	0.77	0.41-1.42	0.41
Body mass index	1.03	0.97-1.08	0.30	1.05	0.96-1.16	0.24
Systolic blood pressure	0.99	0.96-1.02	0.53	1.01	0.97-1.05	0.41
						
Diastolic blood pressure	0.99	0.94-1.04	0.81	1.05	0.99-1.11	0.09
						
Glucose	0.99	0.98-1.00	0.42	1.01	0.94-1.02	0.21
Total cholesterol	0.99	0.98-1.00	0.29	1.01	0.99-1.02	0.31
						
HDL cholesterol	0.97	0.93-1.02	0.30	1.05	0.97-1.09	0.09
						
LDL cholesterol	0.99	0.98-1.01	0.49	1.02	0.97-1.04	0.85
						
Triglycerides	0.99	0.9901.00	0.51	1.04	0.99-1.05	0.25
						
Urea	1.01	0.98-1.02	0.58	1.02	0.97-1.07	0.39
						
Creatinine	0.79	0.30-2.11	0.64	1.82	0.18-17.98	0.61
CAD	0.29	0.09-0.95	**0.04**	0.45	0.05-3.90	0.47
Retinopathy	0.94	0.35-2.49	0.91	1.62	0.38-6.93	0.51
Hypertension	1.53	0.31-7.53	0.59	0.62	0.14-2.71	0.53
Hyperlipidemia	1.64	0.49-5.42	0.41	0.68	0.12-3.60	0.65

## Discussion

It is well documented that T2D patients showed increased prevalence of ASB [4-7]. The novel finding of the present study was that diabetic subjects with MA showed high prevalence of ASB compared to diabetics without MA. This result is in accordance with findings of two recent studies [16,17]. The first study showed that macroalbuminuria and serum creatinine were independent and significant risk factors for ASB in T2D women [16]. The second study, similarly, showed that the main risk factors for ASB in diabetic patients were female gender and urinary albumin excretion [17]. The same observation was made in type 1 diabetic (T1D) subjects. A study in children and adolescents with T1D showed that microalbuminuria and microvascular complications were significant risk factors for ASB [18]. In T1D women ASB was related with the presence of macroalbuminuria [7]. Therefore, a simply explanation might be that albuminuria, as an expression of structural damage in the kidney, might increase the vulnerability to bacterial attacks, thus resulting in an increased risk of developing ASB [7].

In the present study the most common isolated pathogen was *Escherichia coli *in diabetic subjects with and without MA. Previous studies have showed that *Escherichia coli *is the most common pathogen in diabetic subjects either with ASB or with clinical manifestations of UTIs [19,20]. However, one study had different results showing that *Klebsiella spp *was the most common pathogen in T2D subjects with ASB [21]. In the present study *Klebsiella spp *was the third in row isolated pathogen in both groups.

Confirming previous results [5-7] we showed that in the diabetic group with MA presence of ASB was related with female gender. Further analysis of our data showed a relationship between ASB and CAD in the diabetic study group with MA. A simply explanation is that the group with MA had more CAD subjects than the group without MA and this might in part explain the above finding. However, a study in outpatient women showed higher prevalence of CAD among women with ASB in comparison with women without ASB [22].

Despite evidence from previous studies [7,23] we failed to show any relationship between ASB and age as well as BMI. However, other studies were in accordance with our results showing no significant association between ASB and age as well as BMI [16,20,24]. Also, no significant relationships were found between ASB and duration of diabetes, glycemic control and microvascular complications. However, the evidences by the literature are conflicting [6,7,25-27]. A study in T1D women showed that risk factors for ASB were a longer duration of diabetes and the presence of peripheral neuropathy [7]. In addition, presence of longstanding complications, such as peripheral neuropathy and peripheral vascular disease has been showed to be associated with ASB [6,25]. However, these findings could not be confirmed by other studies [26,27]. Finally, in accordance with other studies, our data showed no significant associations between glycemic control, in terms of HbA1c, and ASB [7,8].

Our study has some limitations. First of all, as it is mentioned above, the two study groups were not comparable regarding the prevalence of CAD. In addition, the size of the study sample was rather small to conclude possible associations between ASB and the testing variables. Finally, as data were collected from a referral tertiary center, they cannot be extrapolated to the total diabetic population.

In conclusion, ASB is more prevalent among T2D subjects with MA. Screening for ASB is warranted in diabetic patients especially if pyuria is detected in urine analysis since ASB has been found to be a risk factor for developing symptomatic urinary tract infection [28].

## Competing interests

The authors declare that they have no competing interests.

## Authors' contributions

ID, EM and AK participated in the collection and analysis of urine samples and patient's medical history. AP, AS and ES participated in the design of the study and performed the statistical analysis and drafted the manuscript. SK, SB, DP and SP conceived of the study, and participated in its design and coordination. All authors read and approved the final manuscript.
